# Reduced centrifugation duration improves diagnostic performance of an equine tapeworm egg counting technique

**DOI:** 10.1007/s00436-026-08706-1

**Published:** 2026-06-05

**Authors:** Malena Hughes, Nichol E. Ripley, Mackenzie A. Smith, Carlo Carta, Martin K. Nielsen

**Affiliations:** 1 M.H. Gluck Equine Research Center, Department of Veterinary Science, University of Kentucky, Lexington, KY USA; 2 Department of Natural Sciences, Bluegrass Community and Technical College, Lexington, KY USA; 3 Department of Veterinary Medicine, University of Sassari, Via Vienna 2, Sassari, 07100 Italy; 4 Department of Animal and Veterinary Sciences, Aarhus University, Tjele, Denmark

**Keywords:** *Anoplocephala* spp., Precision, Diagnosis, Shedding

## Abstract

*Anoplocephala perfoliata*, can cause severe health problems in horses. Standard coprological tests have performed poorly for detecting *A. perfoliata*, and while a centrifugation-enhanced flotation technique is superior to other techniques, it is time consuming due to three 10-minute centrifugation steps. This study aimed to evaluate modifications of this technique that reduce the centrifugation durations to 5 and 1 min, respectively. Fecal samples were collected from ten adult *A. perfoliata* egg count positive mares and divided into 9 subsamples to allow for three replicate counts for each of the three protocols. The standard 10-minute centrifugation had a mean coefficient of variation (CV) of 76.8% and a 95% confidence interval of 61.4% − 92.1%, while the 5-min and 1-min protocols had CVs of 68.6% (53.7%-83.4%) and 59.0% (48.7% − 69.4%), respectively. Furthermore, the 10-min protocol yielded 70.7% positive counts compared to 82.9% and 86.2% for the 5-min and 1-min protocols, respectively. The 5-min and 1-min protocols were also superior in egg count magnitude, with significantly higher medians compared to the 10-min protocol. Overall, these data demonstrate that reducing the centrifugation time to one minute will improve test performance for detecting and quantifying equine tapeworm eggs in fecal samples.

## Introduction

*Anoplocephala perfoliata* is the most common equine tapeworm and of particular interest due to its association with ileal colic (Nielsen 2016). This parasite releases gravid proglottids, which disintegrate while being passed through the intestinal tract, releasing the eggs. Treatment failure and apparent anthelmintic resistance to pyrantel and praziquantel have recently been documented in *A. perfoliata* infections (Nielsen 2023; Finnerty et al. 2024). To further monitor this emerging resistance, reliable diagnostics are necessary.

A centrifugation-enhanced egg counting protocol has been developed for detection of anoplocephalid eggs, and has been found to outperform McMaster, Cornell-Wisconsin, and Mini-FLOTAC techniques (Proudman and Edwards 1992; Anderson et al. 2024). However, this technique is time-consuming due to three separate 10-minute centrifugation steps, thus limiting its practical utility. We hypothesized that shortening centrifugation time could be done without sacrificing diagnostic performance.

The aim of this study was to compare performance metrics for the original Proudman and Edwards (1992) protocol with two modified protocols utilizing shorter centrifugation times.

## Materials and methods

### Horses

This study was conducted between January and May 2024 and was approved under the University of Kentucky’s Institutional Animal Care and Use Committee (IACUC) protocol number 2021–3879 (Clinical trial number: not applicable). The study population was naturally infected with *A. perfoliata* and multiple other parasite species as part of the University of Kentucky’s parasitology research herd that has not been treated with anthelmintics since 1979 (Lyons et al. 1990).

Eleven mixed-breed mares between the ages of 4 and 19 were enrolled and maintained on the same pasture for the duration of the study. All eleven mares were pregnant at the beginning of the study, with one mare foaling between the February and March collection, and four mares foaling between the April and May collections. After the first collection, one horse was removed from the study due to unrelated health concerns.

### Fecal sampling and collection periods

A total of four fecal samples were collected per horse, with a sample collected rectally or fresh from the stall floor every four weeks for the duration of the study. Each sample was weighed to ensure a minimum of 360 g of feces, placed in airtight bags, and stored at 4 °C for no more than three weeks prior to processing. Sample IDs were blinded to counting technicians to account for inflated count bias towards known tapeworm shedding status and the order in which each egg counting technique was performed (1-minute, 5-minute, 10-minute) was randomized for each collection period.

### Fecal egg counting technique and modifications

#### Proudman and Edwards standard protocol

Fecal egg counts (FECs) were first performed following the protocol described by Proudman and Edwards (1992). Briefly, 40 g of feces was homogenized with 40 mL of water. This mixture was filtered through two layers of cheesecloth (mesh size: Grade 10–2 mm) and poured into three 15 mL conical tubes that were then centrifuged at 1200 *g* for 10 min. Supernatant was discarded, and tubes were filled halfway with tap water, vortexed to resuspend the formed pellet, filled to the top with water, and vortexed to homogenize. The tubes were centrifuged again at 1200 *g* for 10 min, and the supernatant was discarded. Following this, tubes were filled halfway with a saturated sucrose flotation solution with specific gravity ~ 1.27, vortexed to resuspend the pellet, filled to the top with the sucrose solution, vortexed again, and the tubes were again centrifuged at 1200 *g* for 10 min. After this, a transfer pipet was used to form a slight meniscus at the top of each 15 mL tube using the saturated sugar solution. A 22 mm coverslip was then applied and allowed to passively float tapeworm eggs for 10 min before being removed from the tube and transferred to a microscope slide for manual counting at 100x magnification. All FECs were conducted in triplicate.

#### Modified 5-minute protocol

The Proudman and Edwards (1992) protocol was modified where each centrifugation step was modified to 5 min at 1200 *g*. All other steps remained the same.

#### Modified 1-minute protocol

This method modified the centrifugation steps to 1 min at 1200 *g* with all other details remaining the same.

## Statistical analysis

A total of 369 raw counts were determined on 41 fecal collections over the course of four months, with 123 counts for each method. All statistical analyses were conducted using R (R Core Team, 2021; version 2024.5.1), with the following packages: *tidyverse* (Wickham et al. 2019), *rstatix* (Kassambara 2023), *FSA* (Ogle et al. 2025), *dplyr* (Wickham et al. 2023), *ggpubr* (Kassambara 2025), and *coin* (Hothorn et al. 2006), unless otherwise described.

Egg count was the outcome variable, and initial data assessment included Shapiro-Wilk tests and density plots to evaluate distributional assumptions. Data transformations were explored but were insufficient to normalize distributions. Raw tapeworm egg counts were analyzed using generalized linear mixed models in R (version 2024.5.1) to address repeated measures, clustering, and the non-normal distribution of count data. Models were fit with glmmTMB (Brooks et al. 2017) using egg count per replicate as the response and egg counting protocol as a fixed effect. Random intercepts were included for horse ID and sample ID, with sample ID defined as each horse-by-collection combination to account for non-independence among replicate observations from the same fecal collection. An additional model was fit including storage duration (days) as a covariate. Poisson, negative binomial, and zero-inflated negative binomial mixed models were evaluated, and model fit was compared using Akaike’s Information Criterion. The negative binomial mixed model was selected for inference due to a better fit than the Poisson model and was more parsimonious than the zero-inflated negative binomial model. Model diagnostics, including evaluation of dispersion and zero inflation using simulated residuals, were performed with DHARMa (Hartig 2026). Adjusted marginal means, pairwise protocol contrasts, Tukey-adjusted p-values, and 95% confidence intervals were obtained with emmeans (Lenth and Piaskowski 2026) on the response scale. A secondary model including collection month as an additional fixed effect was fit as a sensitivity analysis to assess whether the protocol effect was robust to seasonal variation.

Precision was assessed with the coefficient of variation (CV), which was calculated with 95% confidence intervals for each subsample. ANOVA tests were used to evaluate CV across egg counting protocols and sample collection months, followed by Tukey’s Honestly Significant Difference for pairwise comparisons. Visualization of residuals and density plots supported assumptions of approximate normality. All results were interpreted at the α = 0.05 level.

## Results and discussion

Precision metrics are presented in Table 1. There were no significant CV differences between protocols (*p* = 0.212), likely due to the relatively low number of eggs observed across all three methods. However, even the lowest CV of 59.0% was much higher than CVs reported for various nematode egg counting techniques (Cain et al. 2020; Ripley et al. 2023), and this suggests room for further improvement.

**Table 1:** Coefficients of variation for each anoplocephalid egg counting protocol

Protocol^a^	Coefficient of Variation (%)	95% Confidence Interval
10 min	76.8	61.4–92.1
5 min	68.6	53.7–83.4
1 min	59.0	48.7–69.4

^a^ Three different versions of the Proudman and Edwards (1992) protocol defined by centrifugation time

**Fig. 1:**
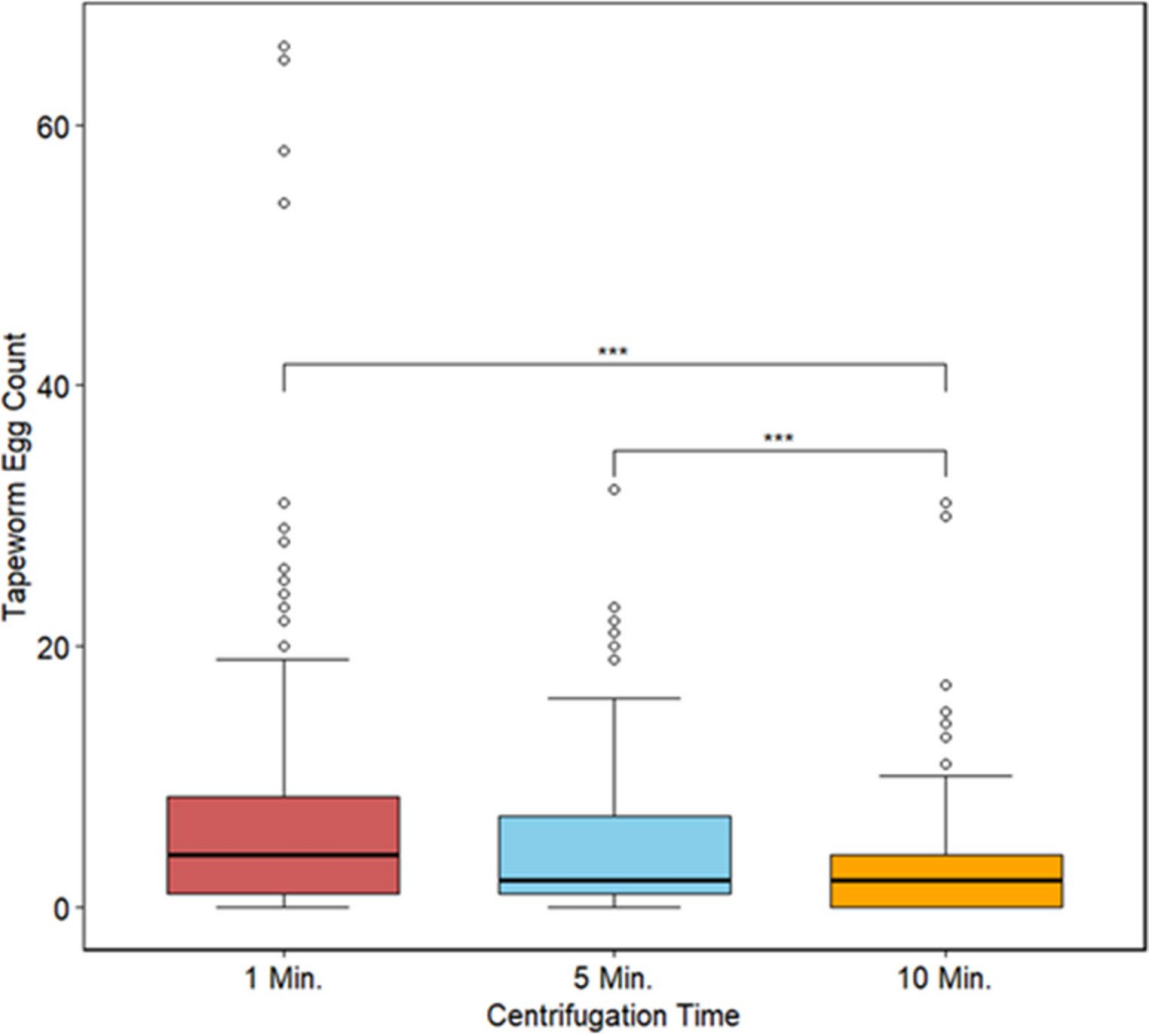
Median anoplocephalid fecal egg counts using three different egg counting protocols defined by three different centrifugation times. *** indicate a significant difference in count magnitude between methods (*p* < 0.05)

The 1-min centrifugation protocol detected the highest proportion of positive samples, with egg recovery reported in 86.2% (106/123) of samples compared to 82.9% (102/123) for the 5-min protocol and only 70.7% (87/123) for the original 10-min protocol. Only 4, 1, and 2 samples were negative on all three replicate counts with the 10-min, 5-min and 1-min protocols, respectively, and in each of these cases samples were positive with both of the other two methods. Table 2 presents the number of negative counts with each protocol that were positive in at least one replicate with the other two protocols. Tapeworm egg count magnitude with each of the three protocols is presented in Fig. 1. As indicated, the 10-min protocol returned significantly lower median counts than both the 5-min (*p* = 0.011) and 1- min protocols (*p* < 0.001). However, effect size was small (η² = 0.043), indicating a modest practical impact. The counts determined by the 5- and 1-min protocols were not statistically different from each other but were trending towards the 1-min being significantly lower (*p* = 0.073). The higher median egg count obtained with the modified protocols is of particular interest due to the emerging need of a standardized fecal egg count reduction test (FECRT) protocol for evaluating anticestodal efficacy. Additionally, the lower proportion of positives returned by the original 10-min protocol suggests that reducing centrifugation time may increase diagnostic sensitivity of this protocol.

**Table 2:** The number of tapeworm egg count negative counts with each of the three evaluated egg counting protocols and the subset that tested positive in at least one of three replicate counts with the two other protocols

		Negative samples testing positive with other protocols		
Protocol^a^	Negative Tests	10 min	5 min	1 min
10 min	36	-	36	33
5 min	21	19	-	18
1 min	17	16	17	-

^a^ Three different versions of the Proudman and Edwards (1992) protocol defined by centrifugation time

A negative binomial GLMM provided a substantially better fit than a Poisson GLMM (AIC 1753.6 vs. 2004.6), whereas adding zero inflation did not improve fit (ZINB AIC 1755.6). In the retained negative binomial GLMM, protocol had a significant effect on egg counts. Adjusted mean counts were 2.03 eggs (95% CI 1.32 to 3.14) for the 10-minute protocol, 3.02 eggs (95% CI 1.97 to 4.63) for the 5-minute protocol, and 4.62 eggs (95% CI 3.02 to 7.06) for the 1-minute protocol. Pairwise comparisons showed significantly lower counts for 10 min versus 5 min (ratio 0.674, *p* = 0.001), 10 min versus 1 min (ratio 0.440, *p* < 0.001), and 5 min versus 1 min (ratio 0.654, *p* < 0.001). These findings were unchanged after adjustment for collection month and storage duration.

In summary, this study suggested that modifying the original Proudman and Edwards protocol to either 1–5 min of centrifugation for each of the three centrifugation steps improves both qualitative and quantitative performance for reasons not understood at this stage. The higher proportion of positive samples suggests improved diagnostic sensitivity, and the significantly higher median egg count suggests improved accuracy. The 1 min protocol appears to be superior to the original 10 min version, and the shorter duration makes it attractive in comparison with the other two protocols.

## Data Availability

All data and materials are available upon request.
